# The association between care modality and hospitalizations and emergency department visits for ambulatory care-sensitive conditions during and after the pandemic in Ontario, Canada

**DOI:** 10.1371/journal.pone.0324805

**Published:** 2025-07-01

**Authors:** Dorsa Mohammadrezaei, Rahim Moineddin, Jun John Wang, Javier Silva Valencia, Maria Carla Lapadula, Angela Ortigoza, Braden O’Neill, Jessica Gronsbell, Debra A. Butt, Anthony Train, Andrea S. Gershon, Karen Tu

**Affiliations:** 1 Toronto Western Family Health Team, University Health Network, Toronto, ON, Canada; 2 Department of Family and Community Medicine, University of Toronto, Toronto, ON, Canada; 3 North York General Hospital, Toronto, ON, Canada; 4 University of British Columbia Hospital, Vancouver Coastal Health, Vancouver, BC, Canada; 5 Vancouver Family Practice Centre, Vancouver, BC, Canada; 6 Departments of Statistical Sciences, University of Toronto, Toronto, ON, Canada; 7 Department of Computer Science, University of Toronto, Toronto, ON, Canada; 8 Department of Family and Community Medicine, Scarborough Health Network, Scarborough, ON, Canada; 9 Department of Family Medicine, Queen’s University, Kingston, ON, Canada; 10 ICES, Toronto, ON, Canada; 11 Sunnybrook Health Sciences Centre, Toronto, ON, Canada; 12 Department of Medicine, University of Toronto, Toronto, ON, Canada; 13 Institute for Health Policy, Management and Evaluation, University of Toronto, Toronto, ON, Canada; University of Porto Faculty of Medicine: Universidade do Porto Faculdade de Medicina, PORTUGAL

## Abstract

The COVID-19 pandemic required a rapid transition to virtual care as a key strategy to maintain healthcare access while minimizing virus transmission risks. However, the impact of this shift on hospitalizations and emergency department (ED) visits for ambulatory care-sensitive conditions (ACSCs) remains unclear. This study aims to assess the relationship between the modality of outpatient care for ACSCs and their outcomes in Ontario, Canada. In this population-based retrospective cohort study, we analyzed hospitalization and ED visit data for ACSCs, including diabetes, epilepsy, congestive heart failure, hypertension, and angina, during the pandemic (April 2020 to April 2023) and post-pandemic (May 2023 to August 2023) periods. Monthly trends in hospitalizations and ED visits were evaluated using Generalized Additive Models and Generalized Additive Mixed Models, accounting for the effects of virtual and in-person care within 30 days and 60 days preceding each event. Despite a notable decrease in virtual visits and a corresponding rise in in-person visits, overall hospitalizations and ED visits for ACSCs remained relatively stable. Our analysis found no significant association between care modality and changes in hospitalizations and ED visits, suggesting that virtual care, particularly during the early pandemic, effectively supported chronic disease management and contributed to the stability of acute care needs. In conclusion, virtual care proved to be a sustainable component of ACSC management during and after the COVID-19 pandemic, complementing in-person care.

## Introduction

The COVID-19 pandemic precipitated a rapid and widespread transformation in healthcare delivery, catalyzing the adoption of virtual care as a crucial modality for maintaining access to healthcare services while reducing the risk of viral transmission [1–4]. In Canada, for example, 75.30% of healthcare visits during the first year of the pandemic were virtual, underscoring the significant shift towards telemedicine during this period [5]. For patients with ambulatory care-sensitive conditions (ACSCs)—chronic diseases such as diabetes, congestive heart failure (CHF), epilepsy, hypertension, and angina—timely outpatient care is vital for reducing the need for hospitalizations or ED visits.

Several studies have investigated the effects of virtual care on ACSC patients, particularly on hospitalizations and emergency department (ED) visits, but most are limited to the first year of the pandemic [6–16]. These studies provide valuable short-term insights but fail to capture the evolving impact of care modalities beyond this initial period. Moreover, the findings have been inconsistent, with some studies reporting a reduction in hospitalizations and ED visits associated with virtual care, while others suggest no significant impact or even an increase in acute care utilization. This variability highlights a critical gap in understanding how virtual and in-person care have influenced outcomes for ACSCs over a more extended period of the pandemic.

Some studies have shown that adopting virtual care could decrease ED visits and hospitalizations for ACSCs. Research from Ontario, for example, found that virtual visits were linked to fewer hospitalizations for conditions such as hypertension, angina, diabetes, and epilepsy in early 2020, though hospitalization rates returned to pre-pandemic levels by mid-2021. Despite this, reductions in hospitalizations for asthma, chronic obstructive pulmonary disease (COPD), and CHF persisted throughout the same period, indicating that virtual care may have contributed to changes in outpatient care trends without an overall increase in hospitalizations for ACSCs [9,10]. Another discovered that patients whose primary care physicians conducted over 20% of their appointments virtually had fewer ED visits than those with a lower percentage of virtual consultations, with a relative rate of 0.77, consistent across both urban and rural settings [7]. Similarly, findings from Alberta suggested that virtual visits were associated with reduced hospitalizations for heart failure (HR: 0.90), hypertension (HR: 0.88), and diabetes (HR: 0.90) within 30 days without negatively impacting follow-up care or short-term outcomes at 30 and 90 days post-visit [11].

On the other hand, some studies highlight potential limitations in virtual care’s ability to reduce hospitalizations or ED visits for ACSCs. Evidence from Michigan primary care practices categorized telehealth use into tertiles, with high telehealth practices (median 39% of visits conducted virtually) showing an increase of 2.10 acute care visits per 1,000 patients annually, compared to low telehealth practices (0.4% of visits). This includes conditions such as diabetes and CHF, suggesting that higher telehealth usage may not effectively reduce hospitalizations or ED visits for these chronic conditions [14]. Additionally, research examining virtual care usage patterns found that while both low and high virtual care users saw reduced hospitalizations during the early pandemic phase, hospitalization rates later increased among high virtual care users. This suggests that the association between care modality and hospitalization may change over time and depend on patient engagement with virtual care [13].

Given the inconsistent results from previous studies and the limited analysis periods, there is a clear need for further research to study the impact of virtual and in-person care on ACSCs over a longer period. While virtual care may also offer cost and resource advantages in managing chronic conditions [17–19], our primary focus here is to determine whether substituting or supplementing in-person visits with virtual care affects hospitalizations and ED visits. This study is the first to examine the association between virtual and in-person care modalities and clinical outcomes for five key ACSCs—diabetes, CHF, epilepsy, hypertension, and angina—across a three-year period (2020–2023). By extending the analysis period, we aim to assess the long-term viability of virtual care as an alternative to, or in combination with, traditional in-person visits for managing ACSCs. The findings of this study will be crucial for informing future healthcare policies and practices in the context of virtual care.

## Methods

### Data sources

This retrospective observational study examines hospitalization and ED visit data for ACSCs and healthcare provider visits in Ontario. Data were sourced from three primary databases: the Canadian Institute for Health Information (CIHI) Discharge Abstract Database (DAD), the National Ambulatory Care Reporting System (NACRS), and the Ontario Health Insurance Plan (OHIP). In this study, the CIHI DAD provided detailed data on all acute care hospitalizations for ACSCs from April 2020 to August 2023. This database includes comprehensive records on patient demographics, diagnoses, interventions, and discharge outcomes for hospital stays across Canada [20]. NACRS was used to collect data on ED visits during the same period. NACRS captures information on patient visits to ED, day surgery, and outpatient clinics, including details on patient demographics, clinical diagnoses, and services provided [21]. The OHIP database provided data on physician billing information, which included details on patient visits to healthcare providers. Population data were obtained from the Statistics Canada website to calculate rates of hospitalization and ED visits [22]. Annual population estimates for Ontario residents aged 0–74 were used as denominators when calculating monthly hospitalization and ED visit rates. The total population for each year was 13,650,806 in 2020, 13,693,308 in 2021, 13,935,798 in 2022, and 14,342,506 in 2023.

We utilized the CIHI Ambulatory Care Sensitive Conditions (ACSC) indicators, which are detailed on CIHI’s ACSC website [23], to extract data on hospitalizations and emergency department visits from CIHI DAD and NACRS. We focused on five specific conditions: diabetes, angina, CHF, hypertension, and epilepsy, categorizing grand mal status and other epileptic convulsions (GMS) under epilepsy and heart failure and pulmonary edema (HFPE) under CHF. Respiratory conditions, including asthma and COPD, were excluded from our study because public health measures during the pandemic—such as physical distancing, lockdowns, and reduced transmission of respiratory viruses—significantly decreased hospitalizations and ED visits for these conditions. This decline, unlike trends seen in other chronic diseases [9], could have introduced bias when evaluating the association of care modalities with outcomes.

### Population

This study included patients who had experienced at least one hospitalization or ED visit mainly due to an ACSC between April 2020 and August 2023. The inclusion criteria, based on CIHI’s ACSC website [23], required that the main reason for the hospitalization or ED visit be an ACSC. Additionally, patients needed to be under the age of 75 upon admission. We excluded hospitalizations involving specific cardiac procedures, records where discharge resulted in death, and those involving newborns, stillbirths, or cadaveric donors. Using patient identifiers, we collected data from the OHIP database on both virtual and in-person office visits, including visits to family medicine and specialist offices, within 30 and 60 days prior to each hospitalization and ED visit. The specific International Classification of Diseases (ICD)-10 codes were utilized to determine ACSC-related conditions, along with other relevant procedure codes and some other criteria, as are provided in Table in S1 Appendix.

### Statistical analysis

Data were analyzed monthly to investigate the association between in-person and virtual visits with hospitalization and ED visits for ACSCs. The primary outcome of interest was the monthly rate of hospitalizations and ED visits per 100,000 population. We employed Generalized Additive Models (GAM) and Generalized Additive Mixed Models (GAMM) depending on the specific requirements of the data, analyzing these rates for each condition separately.

The models included the monthly average number of virtual and in-person visits, calculated within both 30 and 60 days prior to each hospitalization/ED, as main predictors. To enhance the robustness of our findings, we fit separate models for each 30-day and 60-day visit period. A smooth function of time was incorporated to capture non-linear temporal trends. Sex (Female and Male) and age groups in years (0–19, 20–34, 35–49, 50–64, and 65–74) were included as categorical variables to adjust for demographic differences. A Gaussian distribution with an identity link function was utilized to handle the data. For rates that were not symmetric or normally distributed, log or square root transformations were applied.

GAMM models were used when it was necessary to account for serial correlation in the residuals, which was modeled using an autoregressive (AR) process. In cases where serial correlation was not a concern, we applied GAM. Additionally, periodic functions (sine and cosine) with varying periods of 5, 10, 6, or 12 were incorporated when potential seasonality or cyclic patterns were identified in the data. Dummy variables were also employed as needed to account for sharp peaks or troughs in specific months for individual conditions. The detailed specifications for each condition, including the type of model used, the correlation structure, and all predictors, are provided in Tables A and B in S3 Appendix. All analyses were conducted in R.

### Ethics

This study was performed using provincial health administrative data from Ontario Canada. The Ontario Ministry of Health provided these datasets through the Ontario Health Data Platform (OHDP) which was de-identified. The authors were unable to identify individual participants. Given the retrospective nature of this de-identified data and the impracticability of obtaining consent from the whole of the population of Ontario, Research Ethics Board (REB) provided a waiver of consent. Data was accessed from March 20, 2024, to July 11, 2024. The study was approved by the North York General Research Ethics Board (number 0256).

## Results

### Hospitalization

**Fig 1:**
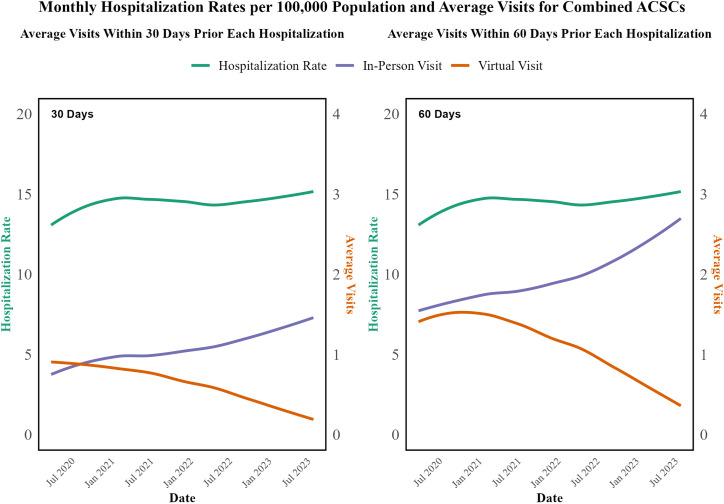
Average Monthly Hospitalization Rates per 100,000 Population and Average In-Person and Virtual Visits within 30 days and 60 days before each hospitalization for Combined ACSCs from April 2020 to August 2023.

**Fig 2:**
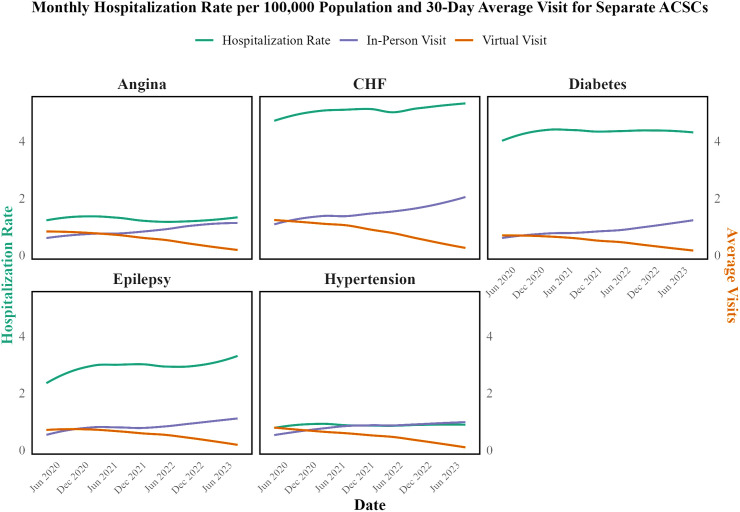
Average Monthly Hospitalization Rates per 100,000 Population and Average In-Person and Virtual Visits within 30 days for Separate ACSCs from April 2020 to August 2023.

As shown in Figs 1 and 2, the trends in hospitalization rates and average visits for both combined and specific ACSCs over time. Despite the substantial decrease in virtual visits and the corresponding increase in in-person visits, the hospitalization rates for ACSCs did not show a consistent pattern of increase or decrease, indicating that the modality of care (virtual vs. in-person) was not significantly associated with the rate of hospitalization for these conditions during the study period. Average monthly hospitalization rates and average in-person and virtual visits within 60 days before each hospitalization for separate ACSCs are illustrated in Fig A in S2 Appendix.

To further investigate this, time series analyses and separate GAMM or GAM were fitted to each condition, as detailed in Table A in S3 Appendix in the Supporting Information. The results from these models indicated that neither virtual visits nor in-person visits within 30 days and 60 days before hospitalization showed significant associations with hospitalization rates for the conditions studied. For instance, for diabetes, the estimates indicated that virtual visits were associated with a small decrease in hospitalization rates within the 30-day period (−0.12, p = 0.025), while in-person visits were associated with a slight increase (0.06, p = 0.031). Similarly, for epilepsy, neither virtual visits (−0.02, p = 0.65) nor in-person visits (0.037, p = 0.183) showed a significant association with hospitalization rates during the 30-day period. This pattern held across other conditions, such as CHF, hypertension, and angina, where neither virtual visits nor in-person visits demonstrated significant associations with hospitalization rates at the 0.01 level of significance. The detailed results of these models, including the estimates, p-values, and effective degrees of freedom (edf), are presented in Table A in S3 Appendix.

### Emergency department visit

**Fig 3:**
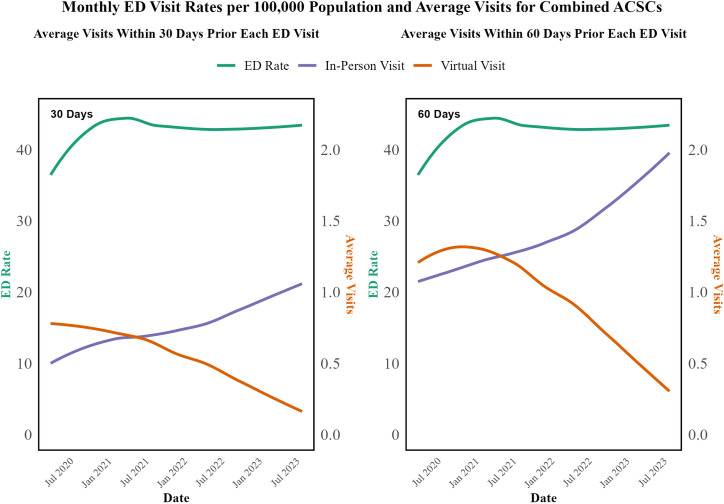
Average Monthly ED Visit Rates per 100,000 Population and Average In-Person and Virtual Visits within 30 days and 60 days before each ED Visit for Combined ACSCs from April 2020 to August 2023.

**Fig 4:**
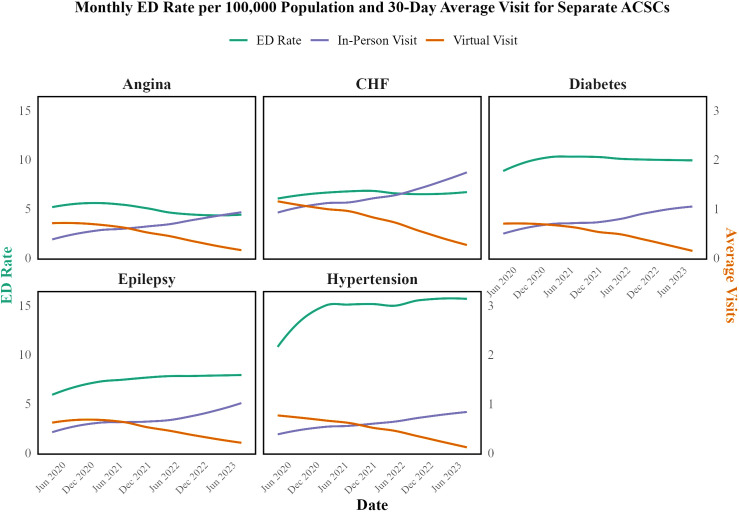
Average Monthly ED Visit Rates per 100,000 Population and Average In-Person and Virtual Visits within 30 days before each ED Visit for Separate ACSCs from April 2020 to August 2023.

Figs 3 and 4 illustrate the trends in ED visit rates and average visits for both combined and specific ACSCs. Despite the significant decrease in virtual visits and the corresponding increase in in-person visits, the ED visit rates for ACSCs showed a relatively stable trend. This similarity to hospitalization trends suggests that the introduction of virtual care did not negatively affect the outcomes for ACSCs. Based on this descriptive analysis, a hypothesis is formed that the modality of care (virtual vs. in-person) may not be significantly associated with the outcome of conditions during the study period. Average monthly ED visit rates and average in-person and virtual visits within 60 days before each ED visit for separate ACSCs are illustrated in Fig B in S2 Appendix.

To further investigate this, time series analyses and separate GAMM or GAM were fitted to each condition, as detailed in Table B in S3 Appendix. The results from these models indicated that neither virtual visits nor in-person visits within 30 days before an ED visit showed significant associations with ED visit rates for the conditions studied. For instance, in the case of epilepsy, virtual visits were associated with a slight, non-significant decrease in ED visits (−0.338, p = 0.247), while in-person visits were associated with a small increase (0.513, p = 0.023) within the 30-day period. For hypertension, virtual visits were associated with a small, non-significant increase in ED visits (0.124, p = 0.071), while in-person visits had no significant effect (0.18, p = 0.642). These findings were consistent across other conditions, such as diabetes, CHF, and angina, where neither virtual visits nor in-person visits demonstrated significant associations with ED visit rates at the 0.01 level of significance. The comprehensive results of these models are presented in Table B in S3 Appendix.

## Discussion

In the early months of the COVID-19 pandemic, hospitalizations, ED visits, and outpatient care for ACSCs declined markedly—by as much as 25–85% for angina, 16% for hypertension, and 4–38% for epilepsy—likely due to lockdowns, social distancing, and public fear [7,9–11,24–32]. [7,9,10] This reduction was coupled with a rise in virtual care, which helped to partially offset the decline in in-person visits, followed by a gradual rebound in care utilization during the later stages of the pandemic [9,33,34].

Our study extends these findings by examining the longer-term trends in hospitalizations and ED visits for ACSCs throughout the COVID-19 pandemic (2020–2022) and into 2023 after the WHO officially declared the pandemic over. Beginning in April 2020, we found a consistent increase in hospitalizations and ED visits for all ACSCs. This trend continued through mid-2021, aligning with the recovery phase observed by Kendzerska et al., where the return to or near pre-pandemic levels for most conditions occurred by March 2021 due to the resumption of delayed care and the easing of pandemic-related restrictions [9].

Following the recovery phase, most conditions—such as diabetes—stabilized with only minimal fluctuations, suggesting the healthcare system adapted to new care modalities. Nonetheless, in early 2023, slight increases were noted in some conditions: hypertension experienced a 20% rise in ED visits and epilepsy a 10% increase in hospitalizations. These modest changes may be due to natural variation or to pandemic-related factors such as heightened stress, lifestyle changes, disruptions in healthcare access, and persistent COVID-19 effects (e.g., systemic hypoxia, inflammation, and abnormal blood coagulation) [35–41].

A key observation from our analysis is that despite dramatic shifts in care modalities—from virtual visits comprising approximately 75% of office visits at the pandemic’s onset to about 20% by August 2023—the overall rates of hospitalizations and ED visits remained consistently stable. Our data indicate that even as virtual visits for ACSCs declined by 69–80% and in-person visits increased by 84.7–169.0%, the transition between care modalities was managed effectively. This suggests that the healthcare system successfully integrated virtual care to maintain the quality of chronic disease management despite changes in delivery mode.

Our statistical analysis also revealed that changes in care modality—whether virtual or in-person—were not significantly associated with increases in acute healthcare incidents, even as overall trends stabilized post-pandemic. This finding confirms the long-term viability of virtual care in managing chronic conditions. During the pandemic, when in-person visits were significantly reduced, virtual care provided a critical alternative, enabling effective management of chronic conditions and preventing the need for acute care. As in-person visits gradually returned to pre-pandemic levels in 2021, the sustained stability in hospitalization and ED visits suggests that the continued use of virtual care was integral in maintaining consistent management of these conditions. This highlights the resilience and adaptability of virtual care as a key component of healthcare delivery, particularly in the management of ACSCs where consistent outpatient care is crucial for avoiding hospitalizations and ED visits [8,9,11,12,42,43].

Virtual care has ensured treatment adherence and chronic illness management, mitigating the impact of reduced access to in-person healthcare services. This is especially beneficial for patients with mobility limitations or those in remote areas, ensuring that even the most vulnerable populations receive necessary care. By facilitating regular follow-ups and consultations without the need for physical visits, virtual care enhances patient engagement, reduces the burden of travel, and causes environmental benefits [44]. Additionally, virtual care offers cost savings for both patients and providers. It eliminates travel expenses and reduces time away from work for patients while lowering overhead costs and administrative burdens for providers by reducing the need for physical infrastructure. These efficiencies enhance accessibility and operational sustainability, making virtual care a cost-effective alternative to in-person visits [18,19]

Despite its advantages, virtual care presents several challenges that must be addressed to optimize its effectiveness. The inability to perform physical exams and detect nonverbal cues can impact diagnostic accuracy, while disparities in digital literacy and access—especially among older adults, economically marginalized populations, and rural communities—continue to limit its reach [8,45,46]. Additionally, the effectiveness of virtual care is limited by challenges in technological infrastructure, with advanced modalities like video consultations being less used due to their higher costs and greater resource demands [44,47–49]. To enhance virtual care effectiveness, healthcare systems should invest in structured provider and patient training, expand digital access initiatives, and implement supportive policies that integrate virtual care into routine practice.

This study contributes to a deeper insight into the association of virtual care with hospitalizations and ED visits for ACSCs during and after the COVID-19 pandemic. It is the first to explore this association over a three-year period, offering valuable insights into the sustainability and effectiveness of virtual care alongside traditional in-person care. Utilizing large-scale, comprehensive data from Ontario, the study conducted robust statistical analyses across multiple chronic conditions, providing a detailed view of how adaptations in healthcare delivery during and after the pandemic influenced patient outcomes over time.

However, several limitations should be considered. While we adjusted for variables such as age and sex, we did not account for other covariates like comorbidity, race, ethnicity, and socioeconomic status, which may influence healthcare access and outcomes. Additionally, our analysis was limited to patients who were hospitalized or visited the ED primarily due to an ACSC condition, which limits the generalizability  of the findings. Comparing ACSC patients who were hospitalized with those who were not could provide more insight into the long-term effects of care modality. Moreover, while we observed trends in outpatient visits and hospitalizations, we lacked detailed information on the specific reasons for outpatient visits, making it difficult to fully understand the context of care. We also lacked data on whether the physician or the patient made the decision for a visit to be virtual or in-person, and we could not assess how factors like the access to the safety equipment or vaccination level influenced the choice of care modality. Finally, our study did not include cost or resource utilization data, limiting our ability to quantify the potential financial benefits or overhead savings of virtual care. Future research should incorporate economic metrics to more comprehensively evaluate virtual care’s overall impact on healthcare delivery.

## Conclusion

This study provides a detailed analysis of the association between the introduction and shifting changes of virtual care and hospitalizations and ED visits for ACSCs during and after the COVID-19 pandemic. Despite a significant reduction in virtual visits and a corresponding rise in in-person care, the overall rates of hospitalizations and ED visits for ACSCs remained largely stable, with only minor fluctuations observed. This stability suggests that the shift from virtual to in-person care did not affect the management of these chronic conditions, underscoring the effectiveness of virtual care as a complementary approach to traditional in-person care.

However, while virtual care proved resilient and adaptable, especially during the pandemic, this study highlights challenges that could be subjects for future research, such as limitations in conducting physical exams, detecting nonverbal cues, and disparities in access due to digital literacy and technological infrastructure. Addressing these challenges is crucial to optimizing virtual care and ensuring its ongoing effectiveness in managing chronic conditions.

## Supporting information

S1 Appendix ICD-10 Codes for ACSCs and Exclusion Criteria. (PDF)

S2 Appendix Monthly Hospitalization and ED Visit Rates with Preceding Visits. (PDF)

S3 Appendix Summary of Statistical Models. (PDF)
