# Supplementary material for: The association between care modality and hospitalizations and emergency department visits for ambulatory care-sensitive conditions during and after the pandemic in Ontario, Canada
Source: PLoS One. 2025 Jul 1;20(7):e0324805. doi: 10.1371/journal.pone.0324805 (PMC12212508; doi:10.1371/journal.pone.0324805)
Supplement: S1 Appendix — (PDF) [file pone.0324805.s001.pdf]

S1 Appendix

ICD-10 Codes for ACSCs

The table below lists the ICD-10 codes used to identify ACSCs included in this study. The ICD-10 is the tenth edition of the ICD. The table also specifies any exclusion criteria related to procedure codes for each condition. For more details on the criteria and methodology used, please refer to the CIHI ACSC indicators [1]

Table S1. ICD-10 Codes for ACSCs and Exclusion Criteria for Procedure Codes.

Condition	ICD-10 Codes	Exclusion-procedure codes
Grand mal status and other epileptic convulsions	G40, G41	None Specified
Diabetes	E10.0, E10.1, E10.63, E10.64, E10.9, E11.0, E11.1, E11.63, E11.64, E11.9, E13.0, E13.1, E13.63, E13.64, E13.9, E14.0, E14.1, E14.63, E14.64, E14.9	None Specified
Heart failure and pulmonary edema	J81 (MRDx*), I50 (MRDx), I50 (as diagnosis type (1) when I11 is MRDx)	† Cardiac procedures
Hypertension	I10 (MRDx), I11 as MRDx when I50 as diagnosis type (1) is not present	† Cardiac procedures
Angina	I20, I23.82, I24.0, I24.8, I24.9	† Cardiac procedures

*MRDx stands for the most responsible diagnosis

† **Cardiac procedures**:
CCP: 47^^, 480^–483^, 489.1, 489.9, 492^–495^, 497^, 498^
CCI: 1.HA.58.^^, 1.HA.80.^^, 1.HA.87.^^, 1.HB.53.^^, 1.HB.54.^^, 1.HB.55.^^, 1.HB.87.^^, 1.HD.53.^^, 1.HD.54.^^, 1.HD.55.^^, 1.HH.59.^^, 1.HH.71.^^, 1.HJ.76.^^, 1.HJ.82.^^, 1.HM.57.^^, 1.HM.78.^^, 1.HM.80.^^, 1.HN.71.^^, 1.HN.80.^^, 1.HN.87.^^, 1.HP.76.^^, 1.HP.78.^^, 1.HP.80.^^, 1.HP.82.^^, 1.HP.83.^^, 1.HP.87.^^, 1.HR.71.^^, 1.HR.80.^^, 1.HR.84.^^, 1.HR.87.^^, 1.HS.80.^^, 1.HS.90.^^, 1.HT.80.^^, 1.HT.89.^^, 1.HT.90.^^, 1.HU.80.^^, 1.HU.90.^^, 1.HV.80.^^, 1.HV.90.^^, 1.HW.78.^^, 1.HW.79.^^, 1.HX.71.^^, 1.HX.78.^^, 1.HX.79.^^, 1.HX.80.^^, 1.HX.83.^^, 1.HX.86.^^, 1.HX.87.^^, 1.HY.85.^^, 1.HZ.53 rubric (except 1.HZ.53.LA-KP), 1.HZ.54.^^, 1.HZ.55 rubric (except 1.HZ.55.LA-KP), 1.HZ.56.^^, 1.HZ.57.^^, 1.HZ.59.^^, 1.HZ.80.^^, 1.HZ.85.^^, 1.HZ.87.^^, 1.IF.83.^^, 1.IJ.50.^^, 1.IJ.54.GQ-AZ, 1.IJ.55.^^, 1.IJ.57.^^, 1.IJ.76.^^, 1.IJ.80.^^, 1.IJ.86.^^, 1.IK.50.^^, 1.IK.57.^^, 1.IK.80.^^, 1.IK.87.^^, 1.IN.84.^^, 1.LA.84.^^, 1.LC.84.^^, 1.LD.84.^^, 1.YY.54.LA-NJ, 1.YY.54.LA-FS, 1.YY.54.LA-NM, 1.YY.54.LA-FR, 1.YY.54.LA-FU

Other Exclusions:

- Records with discharge as death (Discharge Disposition Code = 07, 72, 73, 74)
- Newborn, stillbirth, or cadaveric donor records (Admission Category Code = N, R, or S)

References

1. Ambulatory Care Sensitive Conditions Hospitalizations | CIHI. [cited 20 Feb 2025]. Available: https://www.cihi.ca/en/indicators/ambulatory-care-sensitive-conditions-hospitalizations
