# Supplementary material for: The association between care modality and hospitalizations and emergency department visits for ambulatory care-sensitive conditions during and after the pandemic in Ontario, Canada
Source: PLoS One. 2025 Jul 1;20(7):e0324805. doi: 10.1371/journal.pone.0324805 (PMC12212508; doi:10.1371/journal.pone.0324805)

# S2 Appendix

**Fig S2A. Average Monthly Hospitalization Rates per 100,000 Population and Average In-Person and Virtual Visits within 60 days before each Hospitalization for Separate ACSCs from April 2020 to August 2023.**

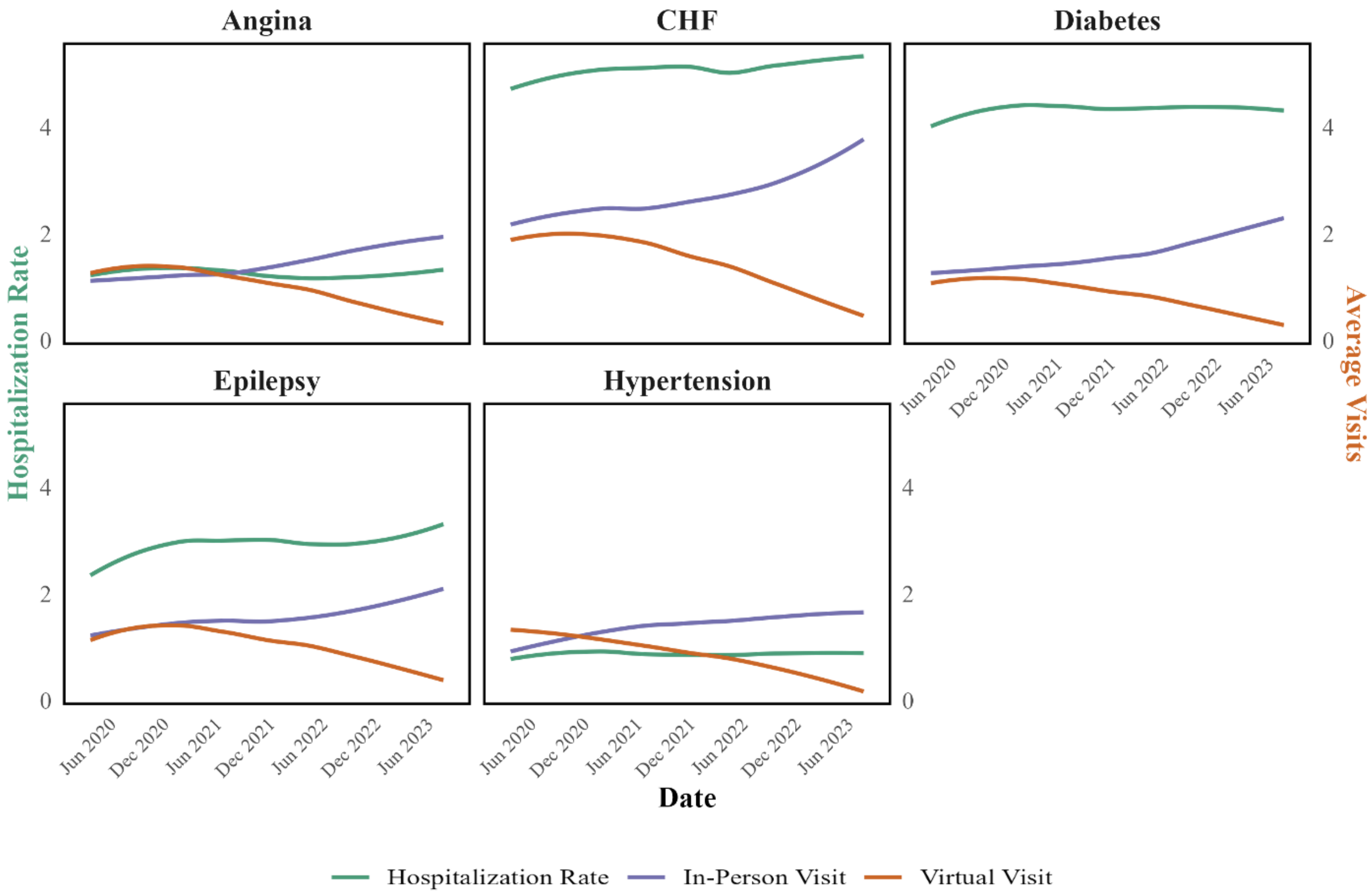

**Fig S2B. Average Monthly ED Visit Rates per 100,000 Population and Average In-Person and Virtual Visits within 60 days before each ED Visit for Separate ACSCs from April 2020 to August 2023.**

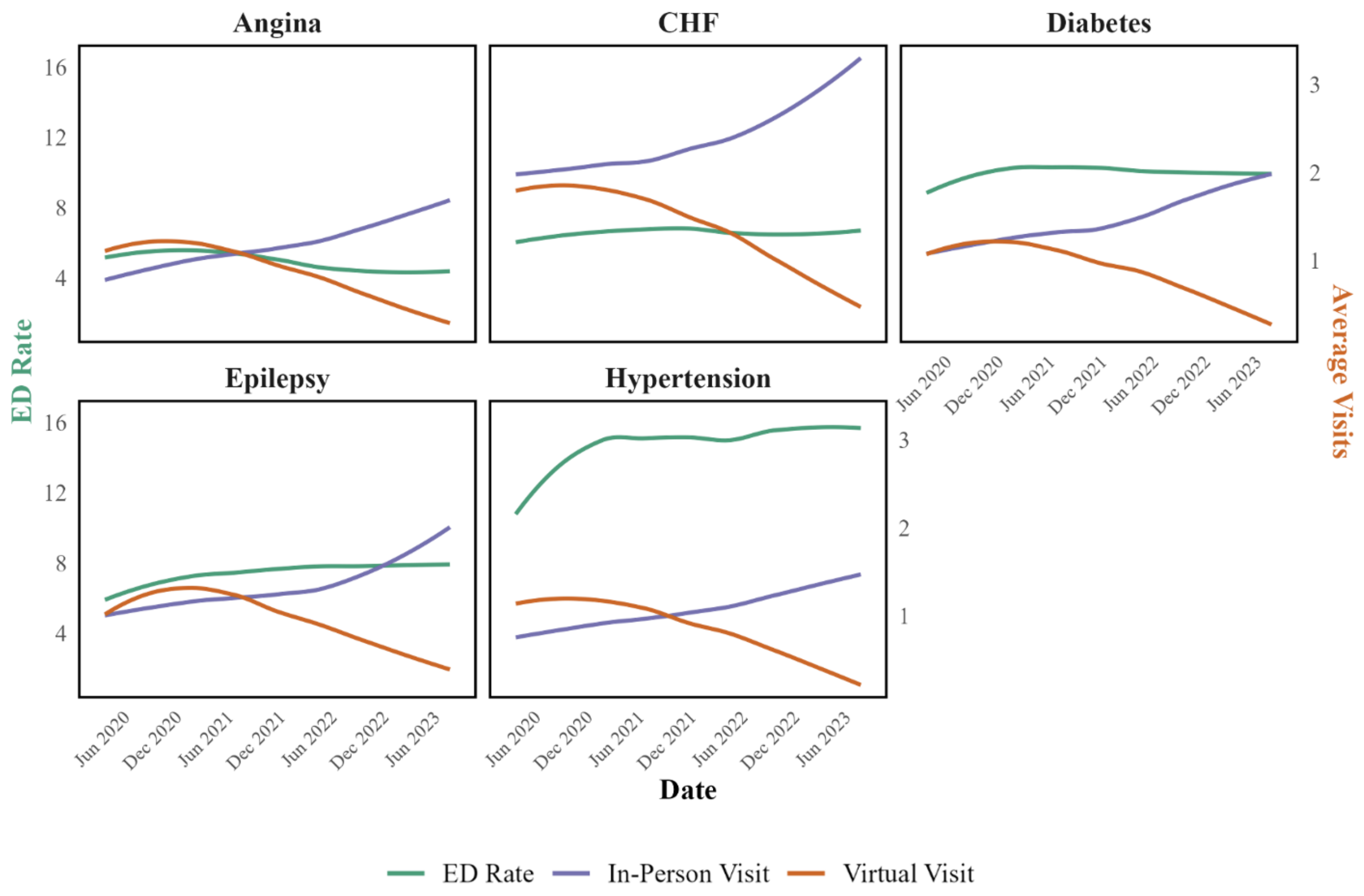

Supplement: S2 Appendix — (PDF) [file pone.0324805.s002.pdf]
