# Supplementary material for: The association between care modality and hospitalizations and emergency department visits for ambulatory care-sensitive conditions during and after the pandemic in Ontario, Canada
Source: PLoS One. 2025 Jul 1;20(7):e0324805. doi: 10.1371/journal.pone.0324805 (PMC12212508; doi:10.1371/journal.pone.0324805)
Supplement: S3 Appendix — (PDF) [file pone.0324805.s003.pdf]

S3 Appendix

Summary of Models

Table S3A. Summary of Models Fitted to Data on Visits within 30 and 60 Days Prior to Hospitalization: Predictors, Outcomes, and Statistical Measures

Diabetes – GAMM Model								
			30-days Period			60-days Period		
**Output**	**Correlation Structure**	**predictors**	**Estimate**	**p-value**	**edf**	**Estimate**	**p-value**	**edf**
Log(Rate Hospitalization)	ARMA(5,0)	Intercept	1	<0.01		0.999	<0.01	
		Virtual visit (avg)	-0.12	0.025		-0.065	0.036	
		In-person visit (avg)	0.06	0.031		0.035	0.038	
		Age group 20-34	0.297	<0.01		0.295	<0.01	
		Age group 35-49	0.37	<0.01		0.369	<0.01	
		Age group 50-64	0.494	<0.01		0.493	<0.01	
		Age group 65-74	0.662	<0.01		0.665	<0.01	
		Gender Male	0.204	<0.01		0.205	<0.01	
		Cos (period 6)	0.005	0.73		0.003	0.837	
		Sin (period 6)	0.021	0.2		0.025	0.137	
		S(time)		0.133	1.835		0.114	1.943
**Epilepsy – GAMM Model**								
Log(Rate Hospitalization)	ARMA(2,0)	Intercept	1.271	<0.01		1.273	<0.01	
		Virtual visit (avg)	-0.02	0.65		-0.003	0.903	
		In-person visit (avg)	0.037	0.183		0.014	0.45	
		Age group 20-34	-0.447	<0.01		-0.448	<0.01	
		Age group 35-49	-0.701	<0.01		-0.702	<0.01	
		Age group 50-64	-0.529	<0.01		-0.527	<0.01	
		Age group 65-74	-0.348	<0.01		-0.345	<0.01	
		Gender Male	0.285	<0.01		0.284	<0.01	
		Cos (period 5)	0.018	0.325		0.019	0.299	
		Sin (period 5)	0.037	0.027		0.037	0.027	
		S(time)		<0.01	3.716		<0.01	3.781

**CHF – GAMM Model**							
Sqr(Rate Hospitalization)	ARMA(5,0)	Intercept	0.079	0.036		0.07	0.058	
		Virtual visit (avg)	0	0.981		0.01	0.46	
		In-person visit (avg)	0.011	0.292		0.006	0.305	
		Age group 20-34	0.258	<0.01		0.252	<0.01	
		Age group 35-49	0.985	<0.01		0.981	<0.01	
		Age group 50-64	2.463	<0.01		2.457	<0.01	
		Age group 65-74	4.692	<0.01		4.684	<0.01	
		Gender Male	0.511	<0.01		0.509	<0.01	
		Cos (period 5)	-0.003	0.86		-0.004	0.809	
		Sin (period 5)	0.015	0.288		0.015	0.277	
		Cos (period 10)	0.023	0.136		0.024	0.127	
		Sin (period 10)	-0.011	0.4		-0.01	0.439	
		S(time)		0.035	1		<0.01	1
**Hypertension – GAM Model**							
Sqr(Rate Hospitalization)		Intercept	0.349	<0.01		0.349	<0.01	
		Virtual visit (avg)	-0.024	0.162		-0.015	0.207	
		In-person visit (avg)	0	0.996		0	0.978	
		Age group 20-34	0.147	<0.01		0.145	<0.01	
		Age group 35-49	0.602	<0.01		0.603	<0.01	
		Age group 50-64	0.906	<0.01		0.907	<0.01	
		Age group 65-74	1.168	<0.01		1.169	<0.01	
		Gender Male	0.027	0.127		0.027	0.14	
		Cos (period 5)	0.016	0.274		0.016	0.265	
		Sin (period 5)	0.026	0.092		0.022	0.098	
		S(time)		0.135	5.434		0.118	5.533
**Angina – GAM Model**							
Log(Rate Hospitalization)		Intercept	-3.163	<0.01		-3.187	<0.01	
		Virtual visit (avg)	0.014	0.799		0.046	0.211	
		In-person visit (avg)	0.026	0.554		0.028	0.336	
		Age group 20-34	0.384	0.024		0.359	0.033	
		Age group 35-49	2.143	<0.01		2.103	<0.01	

		Age group 50-64	3.704	<0.01		3.669	<0.01	
		Age group 65-74	4.351	<0.01		4.309	<0.01	
		Gender Male	0.631	<0.01		0.643	<0.01	
		S(time)		0.369	3.952		0.489	3.932

Table S3B. Summary of Models Fitted to Data on Visits within 30 and 60 Days Prior to ED Visit: Predictors, Outcomes, and Statistical Measures.

**Diabetes – GAMM Model**								
			30-days Period			60-days Period		
**Output**	**Correlation Structure**	**predictors**	**Estimate**	**p-value**	**edf**	**Estimate**	**p-value**	**edf**
Log(Rate_ED)	ARMA(5,0)	Intercept	1.35	<0.01		1.36	<0.01	
		Virtual visit (avg)	0.084	0.056		0.066	0.03	
		In-person visit (avg)	0.082	0.042		0.026	0.272	
		Age group 20-34	0.493	<0.01		0.487	<0.01	
		Age group 35-49	0.77	<0.01		0.766	<0.01	
		Age group 50-64	1.061	<0.01		1.057	<0.01	
		Age group 65-74	1.166	<0.01		1.167	<0.01	
		Gender Male	0.24	<0.01		0.246	<0.01	
		Cos (period 5)	0.026	0.083		0.016	0.32	
		Sin (period 5)	0.012	0.483		0.027	0.066	
		S(time)		0.025	2.344		<0.01	2.288
**Epilepsy – GAMM Model**								
Rate_ED	ARMA(3,0)	Intercept	6.768	<0.01		6.79	<0.01	
		Virtual visit (avg)	-0.338	0.247		-0.09	0.577	
		In-person visit (avg)	0.513	0.023		0.19	0.156	
		Age group 20-34	1.074	<0.01		1.061	<0.01	
		Age group 35-49	-0.923	<0.01		-0.93	<0.01	
		Age group 50-64	-1.916	<0.01		-1.926	<0.01	
		Age group 65-74	-2.76	<0.01		-2.762	<0.01	
		Gender Male	2.34	<0.01		2.34	<0.01	

		Cos (period 6)	0.131	0.162		0.128	0.175	
		Sin (period 6)	0.427	<0.01		0.44	<0.01	
		Cos (period 12)	0.037	0.568		0.037	0.57	
		Sin (period 12)	0.052	0.34		0.048	0.394	
		S(time)		<0.01	8.243		<0.01	8.258
**CHF – GAMM Model**								
Log(Rate_ED)	ARMA(4,0)	Intercept	-2.222	<0.01		-2.22	<0.01	
		Virtual visit (avg)	-0.024	0.571		0.042	0.142	
		In-person visit (avg)	-0.014	0.496		-0.024	0.031	
		Age group 20-34	1.042	<0.01		1	<0.01	
		Age group 35-49	2.792	<0.01		2.75	<0.01	
		Age group 50-64	4.341	<0.01		4.293	<0.01	
		Age group 65-74	5.561	<0.01		5.513	<0.01	
		Gender Male	0.391	<0.01		0.406	<0.01	
		S(time)		<0.01	1		<0.01	1
**Hypertension – GAMM Model**								
Log(Rate_ED)	ARMA(2,0)	Intercept	-1.265	<0.01		-1.261	<0.01	
		Virtual visit (avg)	0.124	0.071		0.087	0.03	
		In-person visit (avg)	0.18	0.642		0.002	0.945	
		Age group 20-34	2.5	<0.01		2.495	<0.01	
		Age group 35-49	3.971	<0.01		3.964	<0.01	
		Age group 50-64	4.56	<0.01		4.55	<0.01	
		Age group 65-74	4.95	<0.01		4.94	<0.01	
		Gender Male	-0.097	<0.01		-0.097	<0.01	
		S(time)		<0.01	7.246		<0.01	7.218
**Angina – GAMM Model**								
Log(Rate_ED)	ARMA(2,0)	Intercept	-2.993	<0.01		-2.976	<0.01	
		Virtual visit (avg)	0.022	0.604		0.027	0.346	
		In-person visit (avg)	0.024	0.655		-0.026	0.384	
		Age group 20-34	1.202	<0.01		1.206	<0.01	
		Age group 35-49	3.55	<0.01		3.56	<0.01	
		Age group 50-64	4.933	<0.01		4.947	<0.01	

		Age group 65-74	5.517	<0.01		5.539	<0.01	
		Gender Male	0.635	<0.01		0.635	<0.01	
		Cos (period 5)	0.062	0.021		0.064	0.016	
		Sin (period 5)	0.016	0.493		0.019	0.416	
		December	0.033	0.618		0.037	0.577	
		May	0.122	0.034		0.127	0.028	
		S(time)		<0.01	3.75		<0.01	3.713
